# Inhibition of testicular embryonal carcinoma cell tumorigenicity by peroxisome proliferator-activated receptor-β/δ- and retinoic acid receptor-dependent mechanisms

**DOI:** 10.18632/oncotarget.5415

**Published:** 2015-09-26

**Authors:** Pei-Li Yao, Li Ping Chen, Tomasz P. Dobrzański, Dylan A. Phillips, Bokai Zhu, Boo-Hyon Kang, Frank J. Gonzalez, Jeffrey M. Peters

**Affiliations:** ^1^ Department of Veterinary and Biomedical Sciences, The Center of Molecular Toxicology and Carcinogenesis, The Pennsylvania State University, University Park, Pennsylvania, USA; ^2^ Chemon Nonclinical Research Institute, Nampyeong-ro, Yangji-myeon, Cheoin-gu, Yongin-si, Gyeonggi-do, Korea; ^3^ Laboratory of Metabolism, National Cancer Institute, Bethesda, Maryland, USA

**Keywords:** peroxisome proliferator-activated receptor-β/δ, testicular embryonal carcinoma, retinoic acid receptor, matrix metalloproteinase-2, tumorigenicity

## Abstract

Peroxisome proliferator-activated receptor-β/δ (PPARβ/δ) has important physiological functions in control of cell growth, lipid and glucose homeostasis, differentiation and inflammation. To investigate the role of PPARβ/δ in cancer, stable human testicular embryonal carcinoma cell lines were developed that constitutively express PPARβ/δ. Expression of PPARβ/δ caused enhanced activation of the receptor, and this significantly decreased proliferation, migration, invasion, anchorage-independent growth, and also reduced tumor mass and volume of ectopic xenografts derived from NT2/D1 cells compared to controls. The changes observed in xenografts were associated with decreased PPARβ/δ-dependent expression of proliferating cell nuclear antigen and octamer-binding transcription factor-3/4, suggesting suppressed tumor proliferation and induction of differentiation. Inhibition of migration and invasion was mediated by PPARβ/δ competing with formation of the retinoic acid receptor (RAR)/retinoid X receptor (RXR) complex, resulting in attenuation of RARα-dependent matrix metalloproteinase-2 expression and activity. These results demonstrate that PPARβ/δ mediates attenuation of human testicular embryonal carcinoma cell progression through a novel RAR-dependent mechanism and suggest that activation of PPARβ/δ inhibits RAR/RXR dimerization and represents a new therapeutic strategy.

## INTRODUCTION

Testicular germ cell tumors are prevalent in men 15–40 years of age with genetic heterogeneity [1]. Although high cure rates have been achieved due to early detection and chemotherapy, the mechanisms of tumorigenesis remain elusive and there is continued need to identify novel targets that will allow for more effective prevention and treatment of this disease with reduced side effects [2–4]. Embryonal carcinoma is the most common component of mixed testicular germ cell tumors, which are difficult to diagnose, and are highly metastatic [5]. Moreover, testicular cancer is associated with other common testicular disorders, including impaired spermatogenesis, cryptorchidism and hypospadias, that likely arise from shared mechanisms [6]. Thus, there is a pressing need to gain new insights into the mechanisms and etiology of testicular cancer development, in particular embryonal carcinomas.

Peroxisome proliferator-activated receptor-β/δ (PPARβ/δ) is a ligand activated transcription factor that actively regulates gene expression through multiple mechanisms [7]. PPARβ/δ and its heterodimerization partner retinoid X receptor (RXR) dynamically bind as a complex to response elements on chromatin after interacting with other proteins such as co-repressors, co-activators, and endogenous agonists [7]. Binding of PPARβ/δ/RXR regulates the expression of a broad range of genes and controls numerous biological processes, including cell differentiation and proliferation, lipid and glucose homeostasis, and inflammation [8]. Interestingly, the relative expression levels and/or presence (or absence) of intra-nuclear co-repressors, co-activators, endogenous agonists, and RXR can influence the activities of nuclear receptors including PPARs, retinoic acid receptors (RARs), thyroid hormone receptor and vitamin D receptor [9–11]. Thus, there are multiple levels of regulation that can impact nuclear receptor-mediated signaling due to competition for the availability of similar co-factors required for an active heterodimerized transcription factor including PPARβ/δ [7, 12–14].

Previous studies showed that over-expression and/or ligand activation of PPARβ/δ suppresses tumorigenicity in skin, colon and breast cancer models [15–17], but the mechanism mediating these effects remains unclear. Interestingly, four of four (100%) human testicular embryonal carcinomas exhibit non-detectable to low expression of PPARβ/δ as compared to non-transformed tissue [18]. In the present study, a stable human testicular embryonal carcinoma cell lines over-expressing PPARβ/δ was established to investigate the mechanisms underlying the anti-tumorigenic effect of PPARβ/δ using *in vitro* and *in vivo* approaches.

## RESULTS

### PPARβ/δ inhibits proliferation, anchorage-independent cell growth, and MMP2 activity in testicular embryonal carcinoma cells

**Figure 1:**
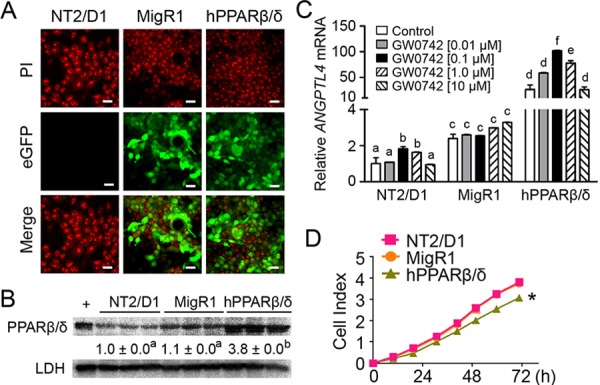
PPARβ/δ inhibits proliferation of human testicular embryonal carcinoma NT2/D1 cells **A.** Representative photomicrographs of NT2/D1, NT2/D1-MigR1 (MigR1, vector control) and NT2/D1-hPPARβ/δ (hPPARβ/δ) cells showing positive eGFP signals in MigR1 and hPPARβ/δ cells. PI staining indicates nuclei. Magnification = 600X. Bar = 10 μm. **B.** Quantitative western blot analysis of PPARβ/δ expression in NT2/D1, MigR1 and hPPARβ/δ cells. +, positive control (cell lysate from COS1 cells transfected with human PPARβ/δ expression vector). Relative PPARβ/δ expression was normalized to LDH. **C.** Relative *ANGPTL4* mRNA expression as compared to NT2/D1 cells with or without the PPARβ/δ agonist GW0742. **D.** Real-time proliferation of NT2/D1, MigR1 and hPPARβ/δ cells. Values represent mean ± S.E.M. Values with different superscript letters are significantly different at *p* ≤ 0.05. *Significantly different than control, *p* ≤ 0.05.

NT2/D1-MigR1 (vector control) and NT2/D1-hPPARβ/δ cells expressed enhanced green fluorescent protein (eGFP), while control NT2/D1 cells were devoid of fluorescence (Figure 1A). Quantitative western blot or qPCR analysis further confirmed that NT2/D1-hPPARβ/δ cells over-expressed PPARβ/δ (Figure 1B), and exhibited enhanced expression of *ANGPTL4* mRNA, a PPARβ/δ target gene, as compared to NT2/D1 parent cells or NT2/D1-MigR1 cells (Figure 1C). Ligand activation of PPARβ/δ with GW0742 robustly enhanced expression of *ANGPTL4* mRNA in NT2/D1-hPPARβ/δ cells compared to controls (Figure 1C). While the higher concentrations of GW0742 did not cause a dose dependent change in *ANGPTL4* mRNA, this is likely due to limited quantity of receptor available for agonist activation, saturation of available receptors, and/or competition with endogenous agonists. NT2/D1-hPPARβ/δ cells exhibited a significant decrease in proliferation compared to controls (Figure 1D). However, no further inhibition of cell proliferation was observed following ligand activation of PPARβ/δ in NT2/D1-hPPARβ/δ cells compared to controls (data not shown).

**Figure 2:**
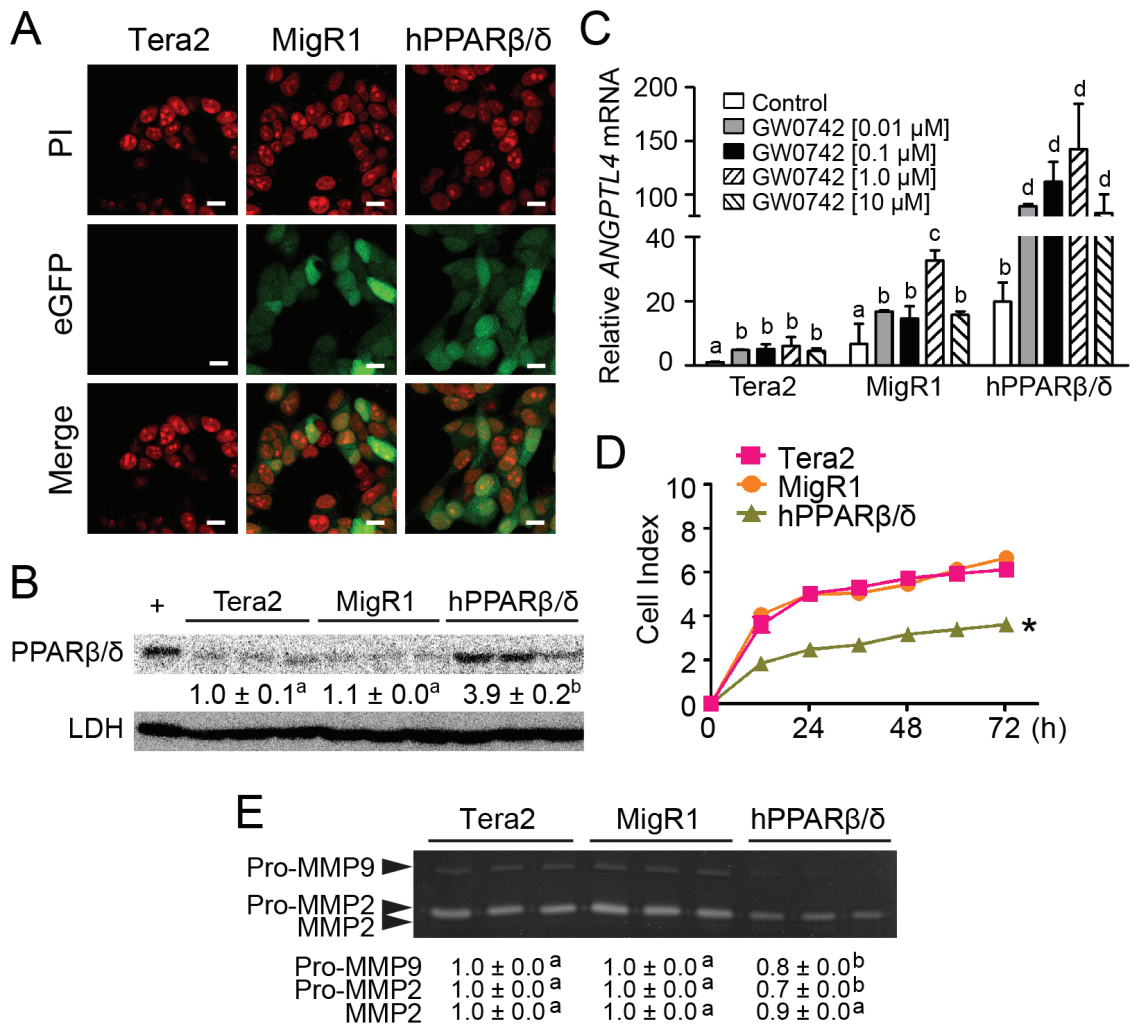
Characterization of human testicular embryonal carcinoma cell line Tera2 over-expressing PPARβ/δ **A.** Representative photomicrographs of Tera2, MigR1 and hPPARβ/δ cells showing positive eGFP signals in MigR1 and hPPARβ/δ cells. PI staining indicates cell nuclei. Magnification = 600X. Bar = 10 μm. **B.** Quantitative western blot analysis of PPARβ/δ expression in Tera2, MigR1 and hPPARβ/δ cells. +, positive control (cell lysate from COS1 cells transfected with human PPARβ/δ expression vector). Relative PPARβ/δ expression was normalized to LDH. **C.** Cells were treated with the PPARβ/δ agonist GW0742 for 24 hours. *ANGPTL4* mRNA expression was determined by qPCR and compared to the parent cell line. **D.** Real-time proliferation was examined in Tera2, MigR1 and hPPARβ/δ cells. **E.** Activities of MMP2 and MMP9 in Tera2, MigR1 and hPPARβ/δ cells were determined by zymography. Values represent mean ± S.E.M. Values with different superscript letters are significantly different at *p* ≤ 0.05. *Significantly different than control, *p* ≤ 0.05.

Another human embryonal carcinoma cell line, Tera2, was also examined. Similar to the results observed with NT2/D1 cells, Tera2 over-expressing PPARβ/δ (Tera2-hPPARβ/δ) and its vector control (Tera2-MigR1) also expressed eGFP, while Tera2 cells showed no fluorescence (Figure 2A). Over-expression of PPARβ/δ in Tera2 cells was confirmed by quantitative western blot analysis (Figure 2B). Higher constitutive expression of *ANGPTL4* mRNA in Tera2-hPPARβ/δ was observed compared to Tera2 cells or Tera2-MigR1 cells (Figure 2C). Enhanced expression of *ANGPTL4* mRNA was also observed following ligand activation of PPARβ/δ by GW0742 (Figure 2C). Over-expression of PPARβ/δ also significantly inhibited cell proliferation compared to controls (Figure 2D). However, no further inhibition of cell proliferation was observed following ligand activation of PPARβ/δ in Tera2-hPPARβ/δ cells compared to controls (data not shown).

**Figure 3:**
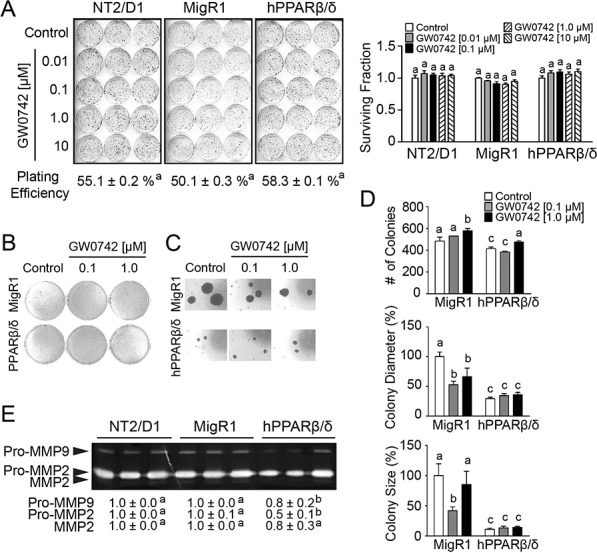
PPARβ/δ inhibits MMP activities and anchorage-independent clonogenicity of human testicular embryonal carcinoma NT2/D1 cells **A.** Left panel, anchorage-dependent clonogenicity of NT2/D1, MigR1 or hPPARβ/δ cells treated with or without GW0742. Right panel, plating efficiency and survival fraction of anchorage-dependent clonogenicity assay. **B.** Anchorage-independent clonogenicity of MigR1 and hPPARβ/δ cells with or without GW0742. **C.** Representative photomicrographs of colonies on soft agar (Magnification = 200X). **D.** Quantification of colonies, diameter of the colonies, and the size of the colonies on soft agar. **E.** Activities of MMP2 and MMP9 in NT2/D1, MigR1 and hPPARβ/δ cells. Values represent the mean ± S.E.M. Values with different superscript letters are significantly different at *p* ≤ 0.05. *Significantly different than control, *p* ≤ 0.05.

Despite the observed inhibition of cell proliferation detected using real-time analysis of NT2/D1 cells over-expressing PPARβ/δ, no difference in anchorage-dependent clonogenicity was observed between NT2/D1, NT2/D1-MigR1, or NT2/D1-hPPARβ/δ cells with or without over-expression and/or ligand activation of PPARβ/δ (Figure 3A). The reason why over-expression of PPARβ/δ caused inhibition of cell proliferation as observed using real-time analysis but had no effect on clonogenicity cannot be determined from these experiments. By contrast, anchorage-independent cell growth was decreased in NT2/D1-hPPARβ/δ cells compared to controls (Figure 3B–3D). MMP activity promotes anchorage-independent transformation [19], and NT2/D1 and Tera2 cells predominately expressed MMP2 (Figure 2E, 3E). Thus, it is of interest to note that MMP2 activity was decreased by 50% and 30% in NT2/D1-hPPARβ/δ and Tera2-hPPARβ/δ cells compared to their controls, respectively (Figure 2E, 3E).

### PPARβ/δ suppresses tumor growth and is associated with reduced cell proliferation and increased necrosis and differentiation

**Figure 4:**
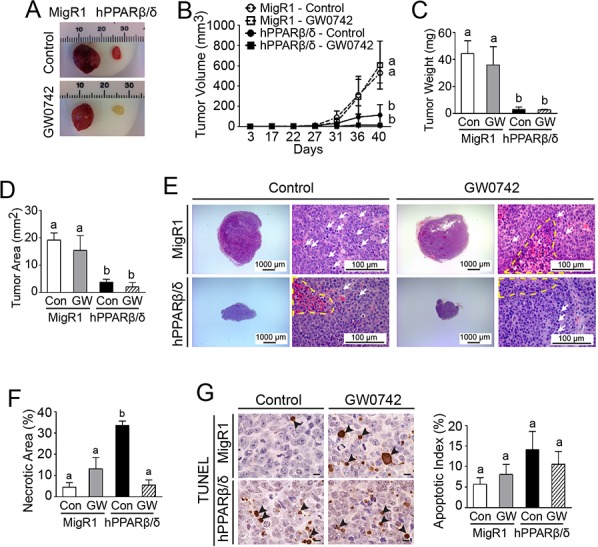
PPARβ/δ attenuates tumor growth in testicular cancer xenografts by inducing necrosis **A.** Representative photomicrographs of xenografts derived from NT2/D1-MigR1 (MigR1) and NT2/D1-hPPARβ/δ (hPPARβ/δ) cells. **B.** Average tumor volumes over time. **C.** Average tumor weight at the end of the study. **D.** Average tumor area in paraffin-embedded tumor sections. **E.** Representative photomicrographs of H&E-stained xenografts. The nuclei of tumor cells were large, pleomorphic and round to spindle, and mitotic figures (white arrows) were frequently observed. Necrotic areas are marked with a yellow dashed line. Magnification = 12.5X (left) and 400X (right). **F.** Average percentage of necrotic area normalized by total tumor area in tumor sections. **G.** Apoptosis in xenograft tumors was determined by TUNEL assay. Left panel, apoptotic tumor cells were indicated by arrowheads. Magnification = 1000X. Bar = 10 μm. Right panel, quantification of apoptotic index in tumor sections. Control = (Con); GW0742-treated (2.5 mg/kg/day) = (GW). Values represent the mean ± S.E.M. Values with different superscript letters are significantly different at *p* ≤ 0.05.

**Figure 5:**
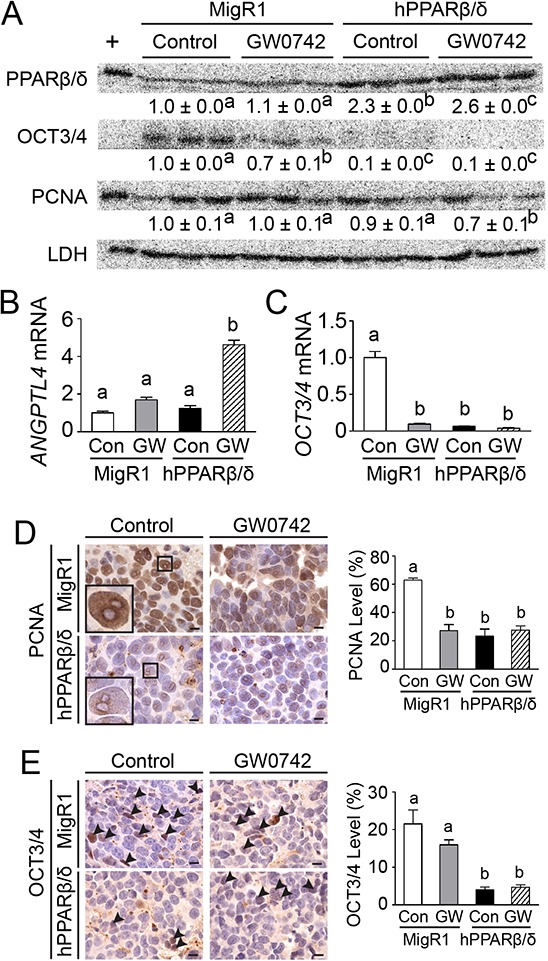
PPARβ/δ induces differentiation and inhibits proliferation in testicular cancer xenografts **A.** the expression of PPARβ/δ, OCT3/4 and PCNA of tumors were determined by quantitative western blot analysis. +, positive control (cell lysate from COS1 cells transfected with human PPARβ/δ expression vector). Relative expression of target protein was normalized to LDH. **B.** *ANGPTL4* and **C.** *OCT3/4* mRNA expression in xenografts. **D, E.** Left panel, representative photomicrographs of xenografts showing the expression of PCNA and OCT3/4 (arrowheads), respectively, assessed by immunohistochemistry. Magnification = 1000X. Bar = 10 μm. D, E. Right panel, quantification of PCNA and OCT3/4 in xenografts. Control = (Con); GW0742-treated (2.5 mg/kg/day) = (GW). Values represent the mean ± S.E.M. Values with different superscript letters are significantly different at *p* ≤ 0.05.

Average tumor volume and weight of ectopic xenografts developing from NT2/D1-hPPARβ/δ cells were markedly smaller compared to controls (Figure 4A–4D). It is worth noting that the tumor incidence was reduced by 40% in mice injected with NT2/D1-hPPARβ/δ cells (6/10) compared to controls (10/10). The incidence of tumor formation was decreased further by ligand activation of PPARβ/δ (4/10) in mice injected with NT2/D1-hPPARβ/δ cells and (6/10) in mice injected with NT2/D1-MigR1 cells. Histopathological analysis indicated that tumor cells of control xenografts were immature and highly mitotic, indicative of malignancy (Figure 4E). In contrast, over-expression and/or ligand activation of PPARβ/δ decreased the size of xenografts compared to controls (Figure 4D and 4E). However, no changes in mitotic indices were noted between groups (date not shown). Interestingly, over-expression of PPARβ/δ caused an increase in necrosis in xenografts from mice injected with NT2/D1-hPPARβ/δ cells, and this effect was not observed in any other group (Figure 4F). A modest increase in apoptosis was observed in xenografts developing from NT2/D1-hPPARβ/δ cells but this effect was not statistically significant (Figure 4G). Further, induced apoptosis in the cell lines was examined in greater detail following treatment with either staurosporine or ultraviolet B (UVB) light exposure. However, no significant changes in poly adenosine diphosphate-ribose polymerase (PARP)-cleavage were observed following staurosporine treatment or UVB exposure among NT2/D1, NT2/D1-MigR1 and NT2/D1-hPPARβ/δ cells (data not shown). Expression of PPARβ/δ was notably higher in xenografts from mice injected with NT2/D1-hPPARβ/δ cells as compared to controls (Figure 5A), and ligand activation of PPARβ/δ caused a marked increase in expression of *ANGPTL4* in xenografts from mice injected with NT2/D1-hPPARβ/δ cells as compared to controls (Figure 5B). Expression of PCNA (a biomarker of proliferation) and OCT3/4 (a biomarker of invasion, migration, and differentiation) was decreased by ligand activation of PPARβ/δ in xenografts from mice injected with NT2/D1-MigR1 cells as compared to controls (Figure 5A and 5C–5E). Moreover, the expression of PCNA and OCT3/4 was decreased by expression and/or ligand activation of PPARβ/δ in xenografts from mice injected with NT2/D1-hPPARβ/δ cells as compared to controls (Figure 5A and 5C–5E).

### PPARβ/δ-dependent attenuation of MMP2-mediated invasion and migration of NT2/D1 cells

**Figure 6:**
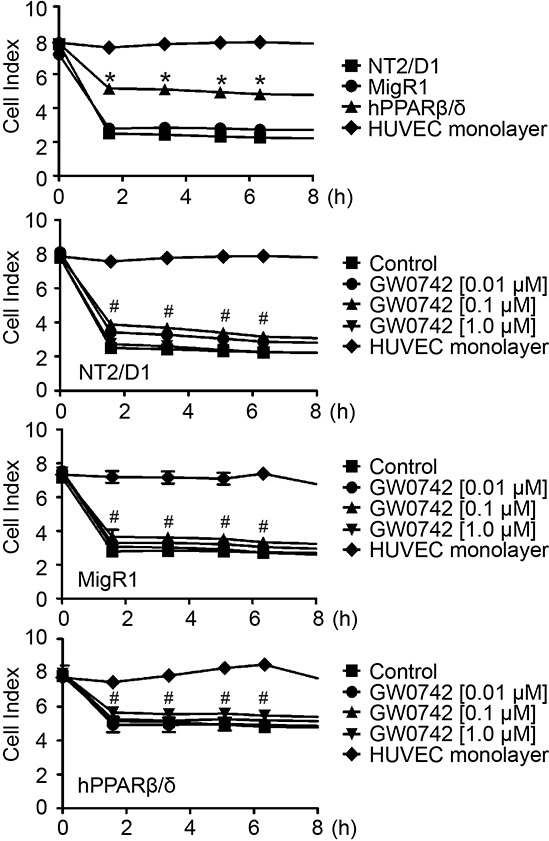
Over-expression of PPARβ/δ decreases testicular cancer cell invasion of a HUVEC monolayer Testicular cancer cell-endothelial cell interaction was detected in real-time using an xCELLigence system. NT2/D1 cell lines were seeded on top of HUVEC monolayer, and cell index was recorded up to 8 hours. Over-expression of PPARβ/δ increased the cell index, indicating a suppression of cell invasion through the HUVEC monolayer. No changes in cell index were observed among NT2/D1 cell lines following GW0742 treatment. Values represent mean ± S.E.M. *Significantly different than NT2/D1 cell control, *p* ≤ 0.05. #Significantly different than the HUVEC negative control, *p* ≤ 0.05.

**Figure 7:**
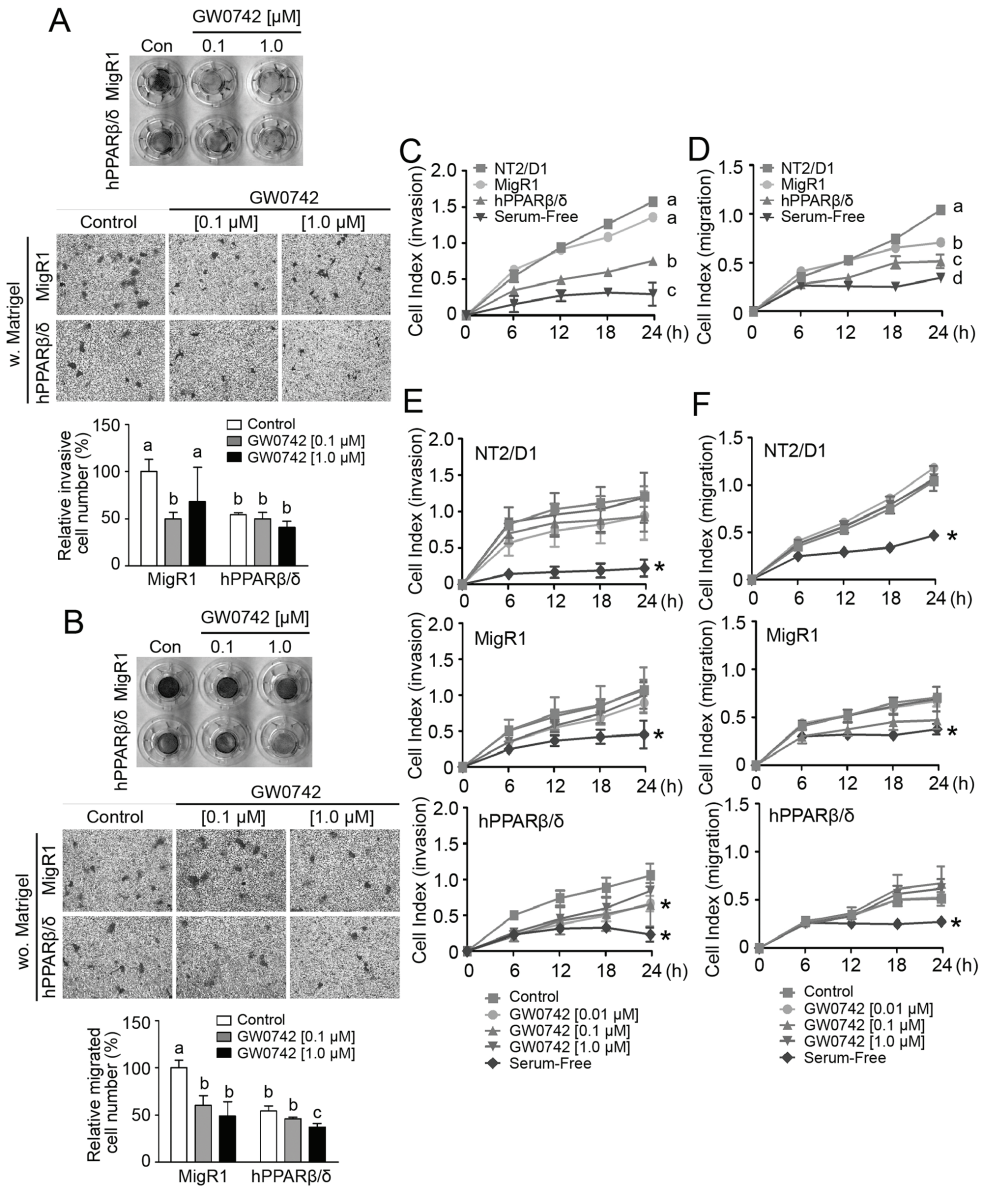
PPARβ/δ inhibits invasion and migration of NT2/D1 cells **A.** Upper panel, representative photographs of transwell inserts in invasion assay. **A.** Middle panel, representative photomicrographs of invasive cells on the lower surface of the transwell inserts. Magnification = 200X. **A.** Lower panel, quantification of invasive cells normalized to NT2/D1-MigR1 (MigR1) control group. **B.** Upper panel, representative photographs of transwell inserts in migration assay. **B.** Middle panel, representative photomicrographs of migrated cells on the lower surface of the transwell inserts. Magnification = 200X. **B.** Lower panel, quantification of migrated cells normalized to MigR1 control group. w: with matrigel; wo: without matrigel. **C, D.** Real-time invasion or migration, respectively, in NT2/D1, MigR1 and NT2/D1-hPPARβ/δ (hPPARβ/δ) cells. **E, F.** Real-time invasion and migration, respectively, of NT2/D1, MigR1 and hPPARβ/δ cells with or without GW0742. Serum-free cells were the negative control. Values represent the mean ± S.E.M. Values with different superscript letters are significantly different at *p* ≤ 0.05. *Significantly different than control, *p* ≤ 0.05.

A real-time invasion assay using human umbilical cord vascular endothelial cells (HUVEC) showed that NT2/D1-hPPARβ/δ cells exhibited weaker invasion through the HUVEC monolayer compared to NT2/D1-MigR1 and NT2/D1 cells, but that ligand activation of PPARβ/δ did not further enhance this effect (Figure 6). In two other transwell assays, over-expression and/or ligand activation of PPARβ/δ in NT2/D1 cells suppressed invasion and migration compared to controls (Figure 7A and 7B). In NT2/D1-hPPARβ/δ cells, invasion and migration as assessed using the real-time xCELLigence system, was reduced as compared to NT2/D1-MigR1 and NT2/D1 cells (Figure 7C and 7D). Inhibition of invasion using the real-time xCELLigence system was also observed in NT2/D1-hPPARβ/δ cells following ligand activation of PPARβ/δ (Figure 7E), but ligand activation of PPARβ/δ had no further influence on migration in any of the cell lines tested (Figure 7F).

**Figure 8:**
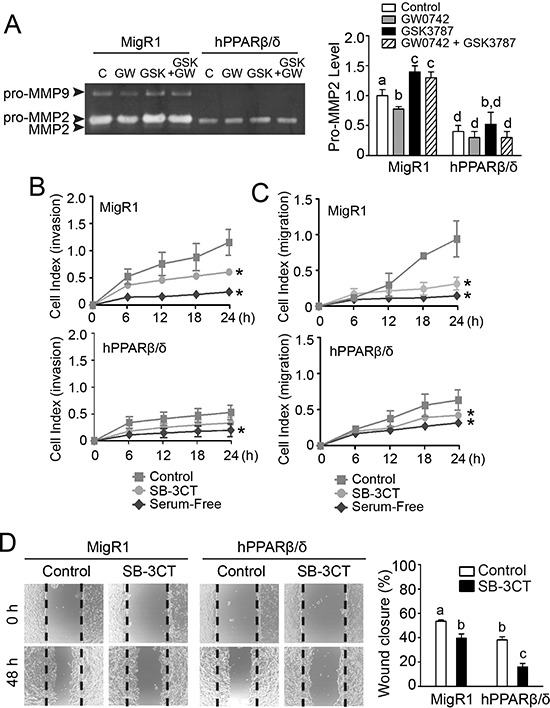
PPARβ/δ-dependent attenuation of MMP2-mediated invasion and migration of NT2/D1 cells **A.** Left panel, activities of MMP2 and MMP9 in NT2/D1-MigR1 (MigR1) and NT2/D1-hPPARβ/δ (hPPARβ/δ) cells treated with vehicle control (C) GW0742 (GW), and/or GSK3787 (GSK). Right panel, relative activity of pro-MMP2. **B, C.** Real-time invasion or migration of MigR1 and hPPARβ/δ cells in response to SB-3CT. **D.** Left panel, representative photomicrographs of wound-healing migration assay in MigR1 and hPPARβ/δ cells in response to SB-3CT treatment and, right panel, the average percentage of wound closure after 48 h. Values represent the mean ± S.E.M. Values with different superscript letters are significantly different at *p* ≤ 0.05. *Significantly different than control, *p* ≤ 0.05.

To determine whether the effects of over-expression and/or ligand activation on invasion and migration required PPARβ/δ, cells were co-treated with and without a PPARβ/δ agonist (GW0742) and/or an antagonist (GSK3787). MMP2 zymographic activity was lower in response to ligand activation of PPARβ/δ with GW0742, and higher in response to the PPARβ/δ antagonist GSK3787 in NT2/D1-MigR1 cells compared to controls (Figure 8A). Co-treatment with GSK3787 and GW0742 mitigated the reduction in MMP2 activity in NT2/D1-MigR1 cells caused by ligand activation of PPARβ/δ alone, compared to controls (Figure 8A). MMP2 zymographic activity in NT2/D1-hPPARβ/δ cells was markedly lower, but ligand activation, treatment with the antagonist or co-treatment with the agonist and antagonist had no further effect on this reduced MMP2 zymographic activity compared to controls (Figure 8A). To demonstrate that MMP2 is required for the PPARβ/δ-dependent changes in invasion and migration, a specific MMP2 inhibitor was examined in these models. Indeed, over-expression of PPARβ/δ in NT2/D1-hPPARβ/δ cells caused a decrease in cell invasion and migration, as assessed by real-time analyses using the xCELLigence system, and it was comparable to that observed with the MMP2 inhibitor SB-3CT (Figure 8B and 8C). Similar results were observed the standard transwell model (data not shown). Further, a wound healing assay showed decreased migration in NT2/D1-hPPARβ/δ cells by treatment with SB-3CT as compared to controls (Figure 8D).

### PPARβ/δ decreases MMP2 activity by competing with RXR and interfering with RAR signaling in NT2/D1 cells

**Figure 9:**
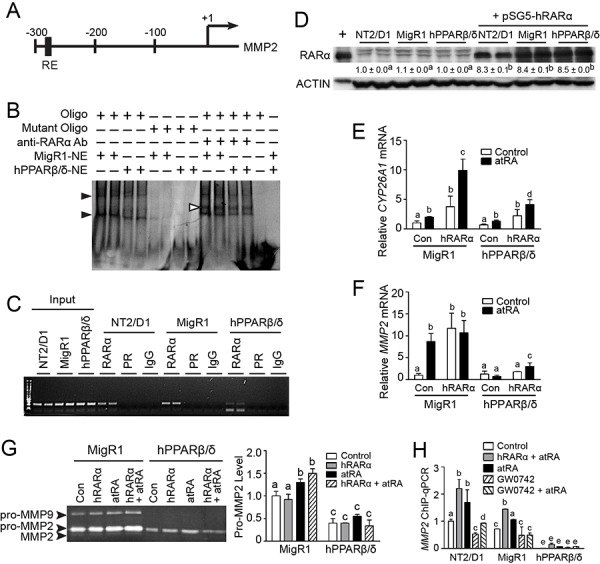
PPARβ/δ suppresses MMP2 activity by interfering with RAR signaling **A.** A putative RAR RE in the human *MMP2* promoter. +1 represented the transcriptional start site. **B.** Representative EMSA of nuclear extracts from NT2/D1-MigR1 (MigR1) and NT2/D1-hPPARβ/δ (hPPARβ/δ) cells were incubated with either double-stranded oligonucleotides (oligo) encoding the RAR RE *MMP2* promoter or mutated oligonucleotides (mutant oligo). Black arrowheads indicated the presence of oligonucleotide-protein complexes. White arrowheads indicated the super shift of oligonucleotide-protein-anti-RAR-antibody complexes. **C.** Representative photomicrograph of the ChIP assay for RAR occupancy on the *MMP2* promoter. **D.** RARα protein expression in NT2/D1, MigR1 and hPPARβ/δ cells transiently transfected with pSG5-RARα plasmid. **E, F.** *CYP26A1* and *MMP2* mRNA expression in MigR1 and hPPARβ/δ cells or cells transiently over-expressing RARα after atRA treatment, respectively. **G.** Left panel, activities of MMP2 and MMP9 in MigR1 and hPPARβ/δ cells transiently over-expressing RAR after atRA treatment, right panel, relative activity of pro-MMP2 in MigR1 and hPPARβ/δ cells. **H.** ChIP-qPCR showing effect of PPARβ/δ on RARα occupancy on *MMP2* promoter in cells over-expressing PPARβ/δ or RARα following atRA and/or GW0742 treatment. Values represent mean ± S.E.M. Values with different superscript letters are significantly different at *p* ≤ 0.05.

The mechanism by which PPARβ/δ regulates MMP2 expression was examined next. The relative availability of different factors including intracellular RXR can influence the activities of PPARs and RARs [10, 12]. A putative RAR RE was identified in the human *MMP2* promoter (Figure 9A). Interestingly, some studies show that atRA can inhibit MMP2 activity [20, 21], while others indicate that atRA can increase MMP2 activity [22, 23]. Thus, the hypothesis that competition between PPARβ/δ and RAR for the availability of RXR required to activate RAR and modulate MMP2 activity was examined. EMSA was performed and two DNA-protein complexes were detected for the putative RAR RE in the *MMP2* promoter (Figure 9B). The binding activities were weaker in the nuclear extracts from NT2/D1-hPPARβ/δ cells compared to that from NT2/D1-MigR1 cells (Figure 9B), suggesting that over-expression of PPARβ/δ competed for RXR binding and reduced the formation of RAR/RXR heterodimers and binding with the *MMP2* promoter. The binding activities were diminished using mutant oligonucleotides, and a super-shift complex was observed in the presence of an anti-RARα antibody for the RAR RE (Figure 9B). Importantly, ChIP assays revealed reduced promoter occupancy of RAR on the *MMP2* promoter in NT2/D1-hPPARβ/δ cells than that in NT2/D1-MigR1 cells (Figure 9C). To more definitively examine the competition between RAR and PPARβ/δ for RXR, NT2/D1 cells were transiently transfected with an RARα expression vector (Figure 9D). The RARα target gene *CYP26A1* and *MMP2* mRNA expression were increased in atRA-treated NT2/D1-MigR1 cells compared to controls, and this induction was greater by over-expressing RARα (Figure 9E and 9F). In contrast, atRA had limited effect on the expression of *CYP26A1* and *MMP2* mRNA in NT2/D1-hPPARβ/δ cells compared to similarly treated NT2/D1-MigR1 cells with over-expression of RARα (Figure 9E and 9F). atRA induced MMP2 activity in NT2/D1-MigR1 cells, and this effect was greater in NT2/D1-MigR1 cells over-expressing RARα compared to controls (Figure 9G). No significant changes in MMP2 activity were detected in NT2/D1-hPPARβ/δ cells over-expressing RARα with or without atRA treatment (Figure 9G). Moreover, ChIP-qPCR demonstrated increased occupancy of RARα on the RAR RE of the *MMP2* promoter following atRA treatment in NT2/D1, and NT2/D1-MigR1 cells, and this induction was attenuated by ligand activation of PPARβ/δ (Figure 9H). Over-expression of PPARβ/δ decreased the occupancy of RARα on *MMP2* promoter in NT2/D1-hPPARβ/δ cells, and the effect of atRA treatment or transient over-expression of RARα on occupancy of RARα on the *MMP2* promoter in NT2/D1-hPPARβ/δ cells was negligible (Figure 9H).

**Figure 10:**
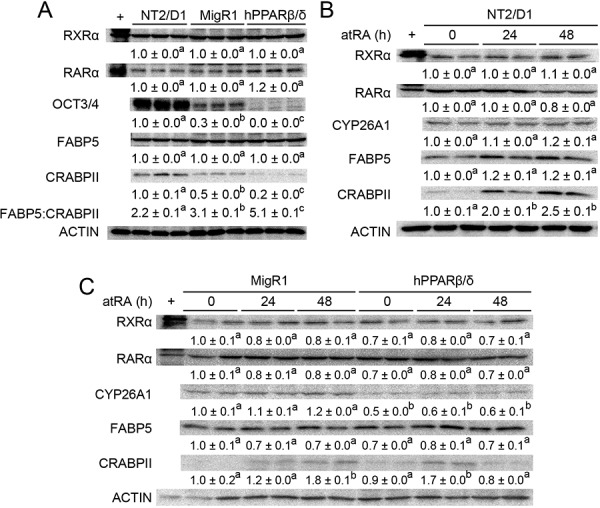
PPARβ/δ interferes with atRA-stimulated signaling in NT2/D1 cells **A.** Quantitative western blot analysis of RXRα, RARα, OCT3/4, FABP5 and CRABPII expression in NT2/D1, NT2/D1-MigR1 (MigR1) and NT2/D1-hPPARβ/δ (hPPARβ/δ) cells. **B, C.** Quantitative western blot analysis of RXRα, RARα, CYP26A1, FABP5 and CRABPII expression in NT2/D1, MigR1 and hPPARβ/δ cells in response to atRA treatment. Values represent mean ± S.E.M. Values with different superscript letters are significantly different at *p* ≤ 0.05.

**Figure 11:**
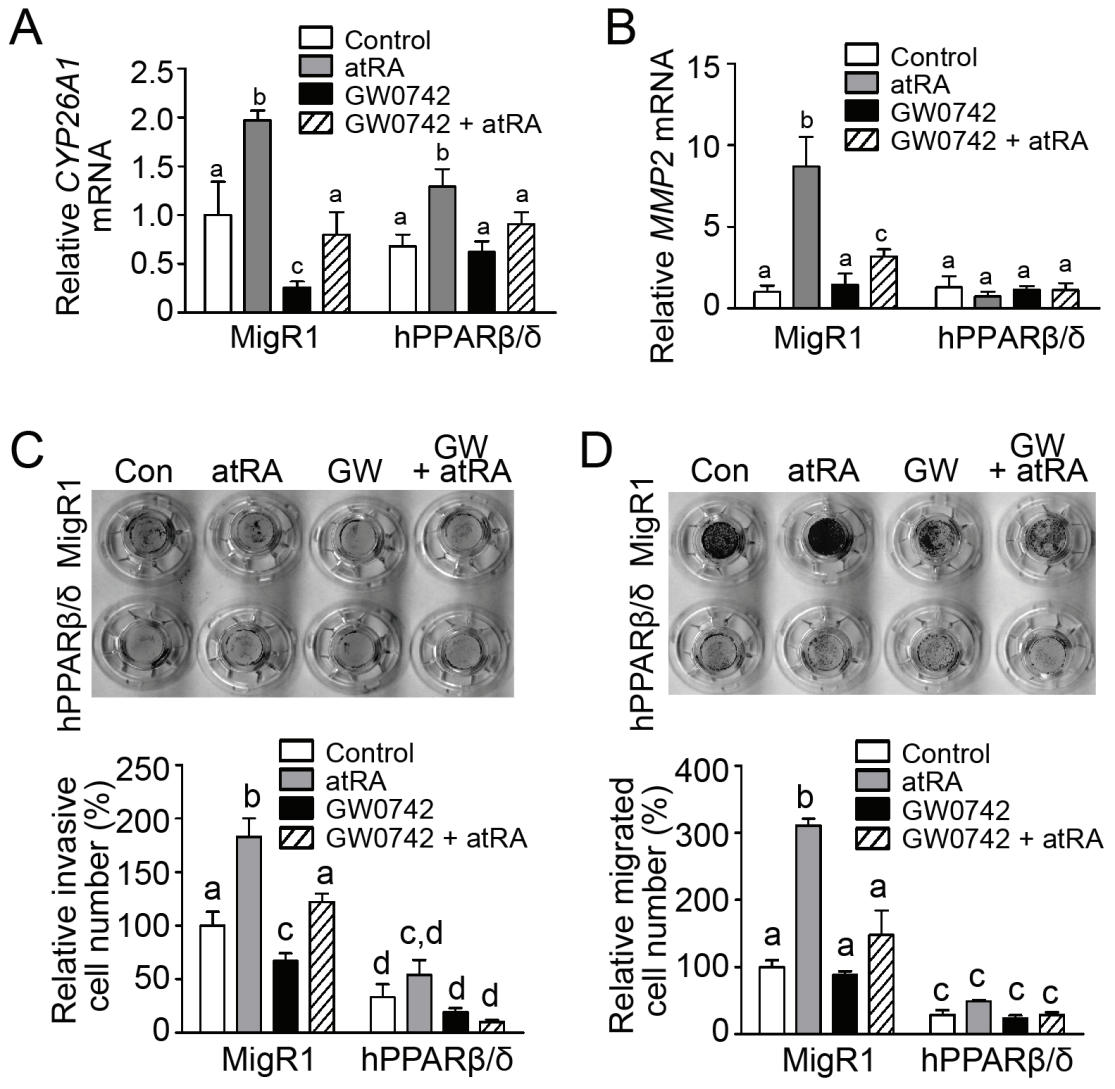
PPARβ/δ interferes with atRA-mediated invasion and migration in NT2/D1 cells **A, B.** *CYP26A1* and *MMP2* mRNA expression in MigR1 and hPPARβ/δ cells following atRA treatment were determined by qPCR, respectively. **C, D.** Upper panel, representative photographs of transwell inserts in invasion or migration assay, respectively, in MigR1 or hPPARβ/δ cells in response to atRA and/or GW0742 treatment, and lower panel, quantification of invasive or migrated cells, respectively, normalized to MigR1 control group. Values represent mean ± S.E.M. Values with different superscript letters are significantly different at *p* ≤ 0.05.

A previous study suggested that atRA can mediate pro-tumorigenic effects in cells exhibiting a relatively high FABP5:CRABPII ratio because the FABP5 putatively “delivered” agonists to PPARβ/δ [24]. Since NT2/D1-hPPARβ/δ cells exhibit a relatively high FABP5:CRABPII ratio compared to control NT2/D1 cells (Figure 10), the effect of atRA on invasion and migration was examined to determine if this mechanism modulated the observed phenotype of the model in the present study (Figure 11). These analyses indicated that atRA signaling mediated by RAR/RXR is not dependent on PPARβ/δ in NT2/D1 cells that exhibit a relatively high FABP5:CRABPII ratio (Figure 10).

## DISCUSSION

Testicular embryonal carcinoma is highly aggressive and metastatic [5]. Interestingly, increasing expression of PPARβ/δ in testicular embryonal carcinoma NT2/D1 cells inhibits anchorage independent transformation, consistent with the observed inhibition of xenograft development from NT2/D1 cells. Results from these studies elucidated two unique mechanisms by which PPARβ/δ inhibits tumorigenesis.

Initiation of testicular cancer is associated with altered differentiation of spermatogonia as they develop stem cell-like properties [25]. The mechanism(s) that mediate this reprogramming is/are not fully understood. Increased expression of OCT3/4 is associated with de-differentiation and induction of pluripotency [26]. Over-expression of PPARβ/δ causes decreased expression of OCT3/4 in xenograft tumors and NT2/D1 cells, suggesting that PPARβ/δ promotes differentiation in these testicular cancer models. Since the induction of terminal differentiation is known to be associated with withdrawal from the cell cycle [27], it is of interest to note that over-expression of PPARβ/δ decreased cell proliferation of NT2/D1 cells and notably in xenografts as revealed by decreased PCNA. Thus, the inhibitory effect of PPARβ/δ on tumorigenicity may be partially linked with PPARβ/δ-induced differentiation modulated by PPARβ/δ-dependent decrease in OCT3/4. However, there remains a need to distinguish whether PPARβ/δ directly triggers cancer cell differentiation and suppresses tumorigenesis, or if it involves developmental reprogramming and causes the reversal of tumorigenesis by decreasing the pluripotency of cancer stem cells. Why expression of PPARβ/δ inhibited proliferation of NT2/D1 cells *in vitro* but ligand activation of PPARβ/δ with a synthetic agonist did not, cannot be determined from these studies. However, the lack of a tumor microenvironment and/or competition with endogenous ligands that may exhibit greater affinity for the receptor could explain this difference.

While relative over-expression of PPARβ/δ is comparable between Tera2 and NT2/D1 cells, the effect on cell proliferation by its over-expression is more robust in Tera2 than in N2/D1 cells. NT2/D1 cells are derived from a single-cell clone of NTERA2 cells originated from xenograft tumors from Tera2 cells in nude mice [28]. Tera-2 cells are highly heterogeneous and consist of only 2–3% of embryonal carcinoma stem cells [29]. Compared to Tera2 cells, NT2/D1 cells represent robust pluripotent embryonal stem cell properties, and are more susceptible to retinoic acid-induced neural differentiation by developing neuronal morphology and exhibiting diminished expression of stem cell makers [29–31]. These differences could contribute to the differential response of NT2/D1 and Tera2 cells on cell proliferation following over-expression of PPARβ/δ. Additionally, NT2/D1 cells also exhibit a similar pattern of DNA methylation as testicular cancer patients at later stage of malignancy [32], suggesting that NT2/D1 cells are more metastatic than Tera2 cells. Thus, these unique characters of NT2/D1 cells allow for examining the role of PPARβ/δ in cancer stem cells and tumor progression.

MMPs promote cancer cell migration, invasion and metastasis. Some studies show that ligand activation of PPARβ/δ increases MMP-dependent cell migration, invasion and metastasis [33, 34], whereas other studies demonstrate that ligand activation of PPARβ/δ inhibits MMP-dependent activities [35–37]. Interestingly, specific repression of PPARβ/δ-dependent gene expression by an inverse agonist (DG172) inhibits human breast cancer cell invasion [38]. The present study used complementary analysis of migration, invasion and MMP expression and activity and revealed strong evidence supporting the prior experiments showing that PPARβ/δ inhibits MMP-dependent activities in testicular carcinoma cells. While there is similar uncertainty regarding the role of atRA in MMP regulation [20, 39–41], results from the present studies also provide strong evidence that RAR can increase MMP2 activity and that increasing expression and activation of PPARβ/δ can inhibit this effect by competing with the heterodimerization partner, RXR, that is used by both RAR and PPARβ/δ. The latter represents a highly novel approach that may be useful for preventing/treating human testicular cancer or other cancers. The utility of increasing PPARβ/δ expression to inhibit RAR-dependent expression of MMP2 and its activity may be feasible since ligand activation of PPARβ/δ can increase expression of PPARβ/δ, possibly through a positive feedback loop [7, 42]. Over-expression of PPARβ/δ did not alter expression of either RXRα or RARα in NT2/D1 cells. However, the expression of CRABPII, a protein involved in the transport of atRA was significantly reduced in NT2/D1-hPPARβ/δ cells. The change in CRABPII expression by PPARβ/δ represents another novel mechanism by which PPARβ/δ may interfere with RAR-dependent MMP signaling. Since atRA caused no change in the FABP5:CRABPII ratio, results from the present study are inconsistent with previous work suggesting that atRA acts as a selective PPARβ/δ agonist [24].

No change in apoptosis was observed in the xenografts in the present study. Further, staurosporine-induced and/or UVB-induced apoptosis was unaffected by ligand activation or over-expression of PPARβ/δ. Combined, this suggests that PPARβ/δ has limited effect on regulating apoptosis in testicular embryonal carcinoma cells. However, increased necrosis was observed in xenograft tumors derived from NT2/D1-hPPARβ/δ cells that was associated with suppression of tumor mass. While ligand activation of PPARβ/δ did not further increase the percentage of necrotic area within the tumor, this is likely due to the extremely small size of these tumors. This is also consistent with studies showing that high expression of PPARβ/δ causes necrosis in colon and breast cancer cell lines [15, 16]. Thus, there is accumulating evidence that PPARβ/δ-induced necrosis may be a common therapeutic/preventive mechanism. Further studies are needed to determine how PPARβ/δ regulates necrosis.

Results from this present study demonstrate that PPARβ/δ attenuates tumor progression in testicular embryonal carcinoma, in part, through interfering with RARα signaling and suppressing MMP2-mediated cell invasion and migration. Whether this regulation is limited in testicular cancer cells or is a common mechanism in other cancer cells remains uncertain. Further detailed investigations are required to examine this idea. This study suggests that specific modulation of RARα signaling may be a viable approach for the treatment of testicular germ cell tumors.

## MATERIALS AND METHODS

### Cell lines

The human testicular embryonal carcinoma cell lines, NTERA-2 cl. D1 (NT2/D1) and Tera2 were purchased from American Type Culture Collection (ATCC, Manassas, VA) in February 2012. The NT2/D1 cells were cultured in Dulbecco's modified Eagle's media (DMEM, Cellgro, Manassas, VA) supplemented with 10% fetal bovine serum (FBS) and 1% penicillin-streptomycin (Invitrogen, Grand Island, NY) at 37°C with 5% carbon dioxide. Tera2 cells were cultured in McCoy's 5A Medium (Lonza Inc., Williamsport, PA) supplemented with 15% FBS and 1% penicillin-streptomycin (Invitrogen, Grand Island, NY) at 37°C with 5% carbon dioxide. The morphology of the cells was consistent with the description provided by ATCC and low passage cells were used for all experiments. ATCC uses multiple approaches to authenticate human cell lines and to rule out both intra- and interspecies contamination.

### Stable human testicular embryonal carcinoma cells over-expressing hPPARβ/δ

A stable human PPARβ/δ (hPPARβ/δ) over-expressing NT2/D1 or cell line was produced using a bi-cistronic retroviral vector (MigR1-eGFP) encoding the human PPARβ/δ cDNA and eGFP that was transduced into NT2/D1 cells as previously described [15]. 1 × 10^4^ control NT2/D1 cells with stably integrated MigR1-eGFP (NT2/D1-MigR1) or NT2/D1 cells over-expressing the bi-cistronic MigR1-eGFP-hPPARβ/δ vector (NT2/D1-hPPARβ/δ) were seeded on 4-well chamber slides (Thermo Scientific, Waltham, MA) and mounted in Vectashield Mounting Medium containing propidium iodide (PI) (Vector Labs, Burlingame, CA). Cells were imaged using Olympus FV1000 laser scanning confocal microscope (Olympus America Inc., Melville, NY) to detect eGFP and PI signals with excitation/emission wavelengths of 499/519 nm and 652/668 nm, respectively. Cells were treated with or without GW0742 (a specific PPARβ/δ agonist [43]), GSK3787 (a specific PPARβ/δ antagonist [44]), or SB-3CT (a specific gelatinase inhibitor [45]). GW0742 and GSK3787 were kindly provided by Drs. Andrew Billin and Timothy Willson (GlaxoSmithKline, Research Triangle Park, NC) and SB-3CT was purchased (Sigma-Aldrich. St. Louis, MO).

A similar strategy using retroviral transduction described for the NT2/D1 cells was performed to generate stable Tera2 cell line over-expressing PPARβ/δ.

### Western blot analysis

Quantitative western blot analysis using a radioactive detection method was performed as previously described [15]. Primary antibodies used included proliferating cell nuclear antigen (PCNA), RXRα, RARα, ACTIN (Santa Cruz Biotechnology, Santa Cruz, CA), cellular retinoic acid-binding protein II (CRABPII), cytochrome P450 26A1 (CYP26A1), PPARβ/δ (Abcam, Cambridge, MA), octamer-binding transcription factor 3/4 (OCT3/4; Cell Signaling Technology, Danvers, MA), fatty acid binding protein 5 (FABP5; BioVedor Inc., Asheville, NC) and lactate dehydrogenase (LDH; Rockland, Gilbertsville, PA), The expression level of each protein was normalized to LDH or ACTIN.

### Quantitative real-time polymerase chain reaction (qPCR)

Expression of target genes in NT2/D1 cells was determined by qPCR analysis as previously described [15]. The forward and reverse primers, respectively, were: angiopoietin-like protein 4 (*ANGPTL4*, NM_139314) 5′-TCA CAG CCT GCA GAC ACA ACT CAA-3′ and 5′-CCA AAC TGG CTT TGC AGA TGC TGA-3′; *OCT3/4* (NM_002701): 5′-CCT GAA GCA GAA GAG GAT CA-3′ and 5′-CCG CAG CTT ACA CAT GTT CT-3′; *CYP26A1* (NM_000783): 5′-TTC TGC AGA TGA AGC GCA GG-3′ and 5′-TTT CGC TGC TTG TGC GAG GA-3′; *MMP2* (NM_004530): 5′-GGA CAC ACT AAA GAA GAT GCA GAA G-3′ and 5′-CGC ATG GTC TCG ATG GTA TTC-3′; glyceraldehyde-3-phosphate dehydrogenase (*GAPDH*, NM_002046): 5′-TGC ACC ACC ACC TGC TTA GC-3′ and 5′-GGC ATG GAC TGT GGT CAT GAG-3′. Each assay included a standard curve and a non-template control that were performed in triplicate. Relative mRNA levels of target genes were normalized to the mRNA level of *GAPDH*.

### Cell proliferation assay

The xCELLigence system (ACEA Biosciences, Inc. San Diego, CA) was used for determining the changes in real-time cell proliferation in NT2/D1 and Tera2 cells as previously described [15].

### Anchorage-dependent and anchorage-independent cell growth

Anchorage-dependent clonogenicity of NT2/D1, NT2/D2-MigR1 and NT2/D1-hPPARβ/δ cells (800 cells/dish) with and without ligand activation of PPARβ/δ agonist was determined as previously described [15]. The plating efficiency and surviving fraction were calculated after 14 days of plating as previously described [46]. Anchorage-independent cell growth of NT2/D1-MigR1 and NT2/D1-hPPARβ/δ cells (7,500 cells/dish) on 0.5% base agar and 0.35% top agarose with and without ligand activation of PPARβ/δ were assessed as previously described [47]. Cell colony numbers were counted after 12 days of plating, and the diameter and surface area of colonies were determined by ImageJ software (Version 1.47c; National Institutes of Health, Bethesda, MD).

### Gelatin zymography

Since MMP activity promotes anchorage-independent transformation [19], gelatinolytic activities of MMP2 and MMP9 in serum-free media collected from NT2/D1, NT2/D1-MigR1 or NT2/D1-hPPARβ/δ cells in response to a PPARβ/δ agonist (GW0742), or a PPARβ/δ antagonist (GSK3787) were assessed by gelatin zymography as previously described [48]. The relative gelatinolytic activities of pro-MMP2, MMP2 and pro-MMP9 were determined using ImageJ software (Version 1.47c; National Institutes of Health, Bethesda, MD).

### Ectopic xenograft tumor assay

Tumor growth of xenografts derived from NT2/D1-MigR1 and NT2/D1-hPPARβ/δ cells (5 × 10^6^) were assessed using 6-week-old female immunodeficient athymic nude (nu/nu) mice (Frederick National Laboratory for Cancer Research, Frederick, MD) as previously described [15]. Groups of mice (*n* = 10) were treated with either vehicle control (0.02% dimethylsulfoxide) or GW0742 (2.5 mg/kg/day) for up to 40 days. At the end of this treatment period, mice were euthanized and tumors excised, weighed, sections were fixed in 10% phosphate buffered formalin overnight and then switched to 75% ethanol. A separate section was snap frozen in liquid nitrogen for protein or mRNA analysis. Fixed tumor sections were processed for staining as previously described [15]. Hematoxylin-eosin (H&E) stained tumor sections were examined by a pathologist.

### Immunohistochemistry

Expression and localization of PCNA and OCT3/4 in ectopic xenograft tumors were determined by immunohistochemistry using 3,3′-diaminobenzidine (DAB) as a substrate and counterstained by hematoxylin as previously described [49]. Twenty fields per sections and two sections per tumor sample were analyzed. Relative expression was determined by normalizing the intensity of DAB to hematoxylin signals using ImageJ software (Version 1.47c).

### Terminal deoxy-nucleotidyl transferase-mediated digoxigenin-dUTP nick end labeling (TUNEL) assay

Apoptotic fragmentation of DNA in paraffin-embedded tumor sections was determined using the ApopTag™ kit (Chemicon, Temecula, CA) following the manufacturer's instructions. Twenty fields per sections and two sections per tumor sample were analyzed. The relative level of apoptosis was determined by normalizing the intensity of DAB to hematoxylin signals using ImageJ software (Version 1.47c).

### Invasion of endothelial cell monolayer by testicular cancer cells

The initial step of metastasis involves the degradation of basement membrane and invasion through vascular endothelial cells [50]. Therefore, the interaction between tumor cells and endothelia cells can be an indicator of cell invasion. To determine whether over-expression and/or activation of PPARβ/δ influenced cell invasion, the interaction between NT2/D1 cell lines and human umbilical vein endothelial cells (HUVEC, purchased from ATCC) was monitored in real-time using xCELLigence as previously described [51]. HUVEC cells (2.5 × 10^4^) were seeded on E-plates to form a monolayer, and NT2/D1 cell lines (1 × 10^4^) were seeded on top of HUVEC monolayer. Testicular embryonal carcinoma cell invasion of HUVEC monolayer was recorded up to 8 hours. Cell index was altered due to the disruption of HUVEC monolayer by the invading testicular cancer cells.

### Invasion assay

The transwell invasion assay was performed as previously described [48]. After 48 hours, cells attached to the lower surface of transwell inserts were counted using a light microscope with 200X magnification. Real-time cell invasion in NT2/D1 cells was also performed using the xCELLigence system following the manufacturer's protocol.

### Migration assay

The transwell migration assay and the wound-healing assay were performed as previously described [48, 52]. After 48 hours, cells attached to the lower surface of the transwell inserts were counted using a light microscope with 200X magnification. Relative wound closure was determined using the wound-healing assay and was calculated with the following formula (initial width (~0.6 mm) minus end width after 48 h of culture)/(initial width (~0.6 mm)) × 100. Real-time cell migration in NT2/D1 cells was also performed using xCELLigence following the manufacturer's protocol.

### Electrophoretic mobility shift assay (EMSA)

A putative RAR response element (RE) was identified in the human *MMP2* promoter using the PROMO program [53]. Oligonucleotides containing the RAR binding sequence designed for EMSA are shown in Table 1. The binding pattern of endogenous RAR from NT2/D1-MigR1 and NT2/D1-hPPARβ/δ cells to the putative RE was determined by non-radioactive electrophoretic mobility shift assay as previously described [54] and analyzed using a gel imaging system (Bio-Rad Laboratories, Inc., Hercules, CA). A super shift assay was performed by adding a primary antibody against RARα to the protein-DNA complexes and incubated at 4°C for additional 30 min.

**Table 1:** Oligonucleotide sequences for EMSA, ChIP and ChIP-qPCR

Name	Sequence (5′ → 3′)
*EMSA*	
Wild-type oligonucleotide	
MMP2-F291	ctgacccccagtcctatctgcc
MMP2-R269	ggcagataggactgggggtcag
Mutant oligonucleotide (capitalized)	
ΔMMP2-F291	ctgaccTTcagtcctatctgcc
ΔMMP2-R269	ggcagataggactgAAggtcag
*ChIP, ChIP-qPCR*	
MMP2-F266	tcctaggctggtcctcactg
MMP2-R173	gaggcactggagaagaaagt

### Transient over-expression and activation of RARα in NT2/D1 cells

NT2/D1, NT2/D1-MigR1 and NT2/D1-hPPARβ/δ (5 × 10^5^) were transiently transfected with 10 μg of a pSG5-RARα plasmid using Lipofectamine LTX reagent (Invitrogen, Grand Island, NY) following the manufacturer's recommended procedures. Transfected cells were treated with or without 10 μM all-trans retinoic acid (atRA, a specific RAR agonist, Sigma-Aldrich, St. Louis, MO) for 24 hours. Over-expression of RARα was confirmed by quantitative western blot analysis, and the expression of the RAR target gene *CYP26A1* was determined by qPCR.

### Chromatin immunoprecipitation (ChIP)

The occupancy of RAR on the *MMP2* promoter in response to over-expression and/or activation of PPARβ/δ, or over-expression and/or activation of RARα was determined by ChIP as previously described [7]. Antibodies against RARα, progesterone receptor (PR), and normal rabbit-IgG (Santa Cruz Biotechnology, Santa Cruz, CA) were individually added to histone-DNA complexes for immunoprecipitation of protein bound chromatin. The antibody against PR, which did not bind to *MMP2* promoter, and rabbit-IgG were used as negative controls. The chromatin fragments were amplified by PCR (26 cycles) using primers flanking the putative RARα binding sequence in *MMP2* promoter shown in Table 1, and resolved on 2% agarose gels. ChIP-qPCR was also performed to quantify relative promoter occupancy. The relative level of RAR-occupied *MMP2* promoter in each group was normalized to input control.

### Statistical analyses

All experimental groups were performed in triplicate and repeated using three independent biological replicates. The data were subjected to Student's *t*-test or a parametric one-way analysis of variance (ANOVA) followed by Tukey test for post hoc comparisons. Statistical significance was considered to be achieved when *p* ≤ 0.05.
